# Industrial Applications of Dinoflagellate Phycotoxins Based on Their Modes of Action: A Review

**DOI:** 10.3390/toxins12120805

**Published:** 2020-12-18

**Authors:** Kichul Cho, Jina Heo, Jinwook Han, Hyun Dae Hong, Hancheol Jeon, Hyun-Ju Hwang, Chang-Yu Hong, Daekyung Kim, Jong Won Han, Kyunghwa Baek

**Affiliations:** 1 Department of Applied Marine Bioresource Science, National Marine Biodiversity Institute of Korea (MABIK), Seocheon-gun, Chungchungnam-do 33662, Korea; kichul.cho@mabik.re.kr (K.C.); hiclow@mabik.re.kr (J.H.); hdhong@mabik.re.kr (H.D.H.); hjeon@mabik.re.kr (H.J.); hjhwang@mabik.re.kr (H.-J.H.); kyunghwabaek@mabik.re.kr (K.B.); 2 Growth Engine Research Department, Chungbuk Research Institute (CRI), Chungju, Chungchungbuk-do 28517, Korea; jaheo514@cri.re.kr; 3 Department of Environmental and Urban Research, Jeju Research Institute, Jeju-si, Jeju-do 63147, Korea; hongchangyu@jri.re.kr; 4 Daegu Center, Korea Basic Science Institute (KBSI), Daegu, Gyeongsangbuk-do 41566, Korea

**Keywords:** harmful algal bloom, dinoflagellate, phycotoxins, industrial application, red tide

## Abstract

Dinoflagellates are an important group of phytoplanktons, characterized by two dissimilar flagella and distinctive features of both plants and animals. Dinoflagellate-generated harmful algal blooms (HABs) and associated damage frequently occur in coastal areas, which are concomitant with increasing eutrophication and climate change derived from anthropogenic waste and atmospheric carbon dioxide, respectively. The severe damage and harmful effects of dinoflagellate phycotoxins in the fishing industry have been recognized over the past few decades, and the management and monitoring of HABs have attracted much attention, leaving aside the industrial application of their valuable toxins. Specific modes of action of the organisms’ toxins can effectively be utilized for producing beneficial materials, such as Botox and other therapeutic agents. This review aims to explore the potential industrial applications of marine dinoflagellate phycotoxins; furthermore, this review focuses on their modes of action and summarizes the available knowledge on them.

## 1. Introduction

Microalgae are photosynthetic microorganisms belonging to diverse phyla [1]. Over the past few decades, several green microalgae, such as *Chlorella* spp., *Scenedesmus* spp., and *Dunaliella* spp., have been recognized as useful bioresources for producing commercial materials, namely cosmetics, pharmaceuticals, dietary supplements, biofuels, and biofertilizers [2,3,4,5,6,7]. In contrast, phycotoxin-producing cyanobacteria, dinoflagellates, and raphidophytes are known to generate frequent harmful algal blooms (HABs), thereby causing severe losses to the fishing industry and aquatic ecosystem [8,9,10,11]. For instance, Lake Erie in the United States is a well-recognized recreational place, but the ecosystem services are under threat owing to cyanobacterial-generated HABs. The annual economic loss of fishing expenditures in Lake Erie was estimated to be approximately USD 2.25–5.58 million during bloom formation [12]. Additionally, massive HABs caused by the dinoflagellate *Karenia mikimotoi* resulted in a mass mortality of abalones, with a loss of over USD 290 million in China [11].

Several factors that cause the death of aquatic organisms have been reported, including direct reactive oxygen species production, phycotoxins, and bioactive fatty acids generation [13]. For instance, the raphidophyte *Chattonella marina* produces superoxide anion via an NAD(P)H oxidase-related mechanism [14], and cyanobacterial species, including *Anabaena* spp. and *Microcystis* spp., produce cellular microcystin, which has previously demonstrated human hepatotoxicity via bioaccumulation in the food chain [15]. Among the algal taxa, dinoflagellate is considered a major HAB-forming group, which causes red tide in coastal areas. Many dinoflagellate species show a mixotrophic nature, practicing both photosynthesis and prey ingestion simultaneously [16]. Furthermore, many species produce phycotoxins, such as saxitoxins (STXs), hemolysins (HL), and yessotoxins (YTX), which exhibit intrinsic modes of action. Although managing and monitoring dinoflagellates has been under the spotlight for the past few decades, industrial applications of phycotoxins have not garnered much attention. Therefore, to improve knowledge of their industrial applications and to focus on application-based research, we have summarized the current findings, specific modes of action, and biotechnological potential of the diverse dinoflagellate phycotoxins in this review.

## 2. Effects of HABs Produced by Dinoflagellates

Dinoflagellates are unicellular eukaryotes belonging to phylum Dinoflagellata. Many of its species cause red tide in coastal areas, which significantly damages aquatic life and causes paralytic shellfish poisoning (PSP), neurotoxic shellfish poisoning (NSP), diarrhetic shellfish poisoning (DSP), and ciguatera in humans worldwide [10,11,17,18,19,20]. There are more than 2000 identified dinoflagellate species, and they exhibit distinct characteristics, including those of the autotrophs, heterotrophs, and mixotrophs [21]. Morphological characteristics of dinoflagellates include two dissimilar flagella arising from the ventral cell side. These organisms are capable of producing diverse phycotoxins and render HAB-derived damage [22]. During the 1990s, *Cochlodinium* spp. (mostly *C. polykrikoides* and *C. fulvescens*) caused damage to the fishing industry with an estimated annual loss of more than USD 100 million in South Korea [23,24]. Out of these, *C. polykrikoides* caused an economic loss of approximately USD 69.5 million in 1995. Moreover, a massive bloom of the dinoflagellate *Karlodinium digitatum* caused approximately USD 32 million damage to the fishing industry of Hong Kong [11]. Additionally, *K. mikimotoi* caused a massive economic detriment of more than USD 290 million to the fishing industry in China in 2012 [11].

Harmful effects of dinoflagellate-generated HABs associated with other organisms have been extensively investigated over the past few decades. For instance, Chen et al. reported that a polar lipid-soluble component derived from *K. mikimotoi* extract inhibited proliferation, disrupted cell membrane, and increased lipid peroxidation (increased malondialdehyde content) in mammalian cells [25]. Further, the addition of *K. concordia* extract induced anesthesia in brine shrimp [26] and the PSP (e.g., STX)-producing *Alexandrium fundyense* consumed by copepods was lethal to fish [27].

However, biotoxins derived from diverse organisms have potential applications. In particular, botulinum toxin produced by the bacterium *Clostridium botulinum* is widely used for the treatment of migraine headaches, muscle spasticity, and other muscle disorders [28,29,30]. Additionally, pufferfish-derived tetrodotoxin is therapeutically used to manage acute heroin withdrawal syndrome and alleviate cancer pain [31,32]. These biotoxins have specific modes of action, and they have potential and extensive industrial applications. Although a lot of phycotoxins derived from dinoflagellates have been extensively studied, their industrial application based on their specific modes of action is still poorly understood. Therefore, we describe the modes of action of the diverse dinoflagellate phycotoxins in the following section.

## 3. Dinoflagellate Phycotoxins and Their Modes of Action

Table 1 summarizes the reported dinoflagellate-produced phycotoxins. *Alexandrium* spp. are considered PSP-producing harmful organisms. The causative paralytic toxins of *Alexandrium* spp. include STX, gonyautoxin (GTX), neosaxitoxin (NSTX), and HL [33,34,35,36]. Among these, STXs are well-known marine algal toxins that block the cellular sodium channel. STX is included in the guanidinium neurotoxin group, sharing the common chemical feature of guanidinium moieties and interacting with voltage-gated sodium channels with high binding affinity and ion flux blockage capacity. This blockage induces the reduced influx of Na^+^ ions into a cell and causes inhibition of the propagation of action potentials in excitable membranes. This process ultimately induces neuromuscular paralysis [37]. The symptoms of PSP induced by STX and its analogue, NSTX, include numbness of the lips and gastrointestinal disorders [35]. Lefebvre et al. [34] reported that measurable levels of STX were detected in both field and cultured *Alexandrium* spp. using a receptor-binding assay and enzyme-linked immunosorbent assay. The structure and analogues of STX were previously well-described by Wiese et al. [38]. *Gymnodinium catenatum* and *Pyrodinium bahamense* produce STXs as well [39,40]. Landsberg et al. [40] reported that the source of STXs detected in pufferfish skin, muscle, and viscera was putatively derived from *P. bahamense*. In addition, Sako et al. [39] purified and characterized sulfotransferase-specific STX analogues from the cytosolic fraction of *G. catenatum*. GTXs influence the mammalian nervous system by binding to site 1 of the α-subunit of the voltage-dependent sodium channel in the postsynaptic membrane, thereby preventing synaptic function [41].

Altered hemolytic activity of *A. peruvianum* under different nutrient ratios indicated the presence of cellular HL [42]. Although the modes of action of dinoflagellate HL are poorly understood and algal species-specific, a possible mechanism could be the hydrolysis of phospholipids and subsequent pore formation in phospholipid bilayers, a mechanism similar to other hemolytic toxins [43,44]. Modes of action of HLs detected in *Amphidinium carterae*, *C. polykrikoides*, *Heterocapsa circularisquama*, *K. mikimotoi*, and *Gonyaulax monilata* are described briefly in Table 1 [36,45,46,47,48].

*Azadinium spinosum* produces azaspiracids (AZA), a group of toxic lipophilic polyether compounds. This toxin caused human intoxication symptoms, such as nausea, vomiting, severe diarrhea, and stomach cramps in a study conducted in the Netherlands [49,50]. AZA was originally believed to be a toxic compound produced by *Protoperidinium crassipes* [51]. However, it was later demonstrated that the toxins in *P. crassipes* were a consequence of its feeding on the dinoflagellate *A. spinosum*, which was in turn reported to be the source of the AZA [52]. This toxin causes damage to the intestinal epithelium, lamina propria, liver, and villi as an acute toxic effect, and causes lung tumors and malignant lymphomas at high concentrations with long-term exposure [53,54,55]. AZAs include more than 30 analogues, and among these, only AZA1, AZA2, and AZA3 are currently regulated in edible shellfish by the European Union through their toxic equivalency factors (TEFs) [56]. It exhibited its action by blocking the human ether-a-go-go-related gene (hERG) potassium channel [57]. AZA interacts with the channel’s central pore (F656) within the S6 transmembrane domain and physically blocks the potassium-conductance pathway of the hERG1 channels [57]. Pelin et al. [58] previously reported that the exposure of immortalized human hepatocyte (IHH) cell line to AZA analogues induced mitochondrial electron transport chain complex-dependent mitochondrial dehydrogenases activity (MDA) in a concentration-dependent manner. The MDA was suppressed in the K^+^, Cl^−^, and Na^+^ free media, and by specific inhibitors of K_ATP_ (glibenclamide), hERG potassium channels (cisapride), Na^+^/K^+^, ATPase (ouabain), and cystic fibrosis transmembrane conductance regulator (CFTR) chloride channels (CFTR(*inh*)-172). These results revealed that the AZA-induced MDA is derived from an imbalance of intracellular levels of K^+^ and Cl^−^ ions [58]. The toxic effects, structure, and analogues of AZA were well-described in previous studies [50,59,60,61,62,63].

Another dinoflagellate genus, *Coolia*, produces cooliatoxin (CTX) and YTX [65,66]. Holmes et al. [65] purified a novel toxin from *C. monotis* isolated from Australia and named it CTX. This toxin is considered a monosulfate polyether analogue of YTX and caused initial blockage of unmyelinated nerves in vitro, as reported by Holmes et al. [65]. Additionally, sulfated polyether analogues of YTX have been detected in *C. malayensis* through chemical analysis using NanoLiquid chromatography–mass spectrometry [66]. YTX is a diarrhea-causing toxin and exhibits its toxicity by activating nifedipine and the SKF-96365 sensitive calcium channel [68], and by decreasing cytosolic 3′,5′-cyclic adenosine monophosphate (cAMP) levels [69]. YTX-producing dinoflagellates include *Gonyaulax spinifera*, *Lingulodinium polyedrum*, and *Protoceratium reticulatum* [80,89]. The structure and analogues of YTX were previously well-described by Paz et al. [96].

*Karlodinium armiger* produces karmitoxin (KTX), which is an amine-containing polyhydroxy-polyene toxin [82]. Although its specific mode of action is not yet identified, ichthyotoxic effects of this toxin toward fish larvae and juveniles have been demonstrated recently [97]. The structure of KTX was previously reported by Rasmussen et al. [82]. Additionally, *Karlodinium* spp. produces karlotoxin (KmTx), which is structurally similar to amphidinols and is the causative toxin for membrane permeabilization [83]. KmTx is produced by *K. armiger* and *K. veneficum*, and its mode of mechanism is the disruption of the cell membrane by specifically binding to cholesterol [82]. The structure and several analogues of KmTx were reported by Van Wagoner et al. [98].

*Dinophysis* is a medium-sized dinoflagellate that produces DSP toxins, including okadaic acid (OA), pectenotoxin (PTX), and dinophysistoxin (DPX) [36,73]. OA is a polyether fatty acid, and its structure is highly similar to that of acanthifolicin [71]. DPXs are considered analogues of OA, whereas PTXs are a type of polyether lactones [99,100]. The mode of action of OA and DPXs is an inhibition of the serine/threonine (Ser/Thr) phosphatases that further induces tumor growth promotion and neuronal cell death [71,72,101,102]. The structures and analogues of OA and DPX were previously reported by Uchida et al. [103] and Fernandez et al. [104]. PTXs demonstrate diverse physiological functions, including inhibiting Ser/Thr phosphatases, depolymerizing actin filaments, and disrupting the actin cytoskeleton [35,36,76,77,78]. According to Espiña et al. [105], marked depolymerization of F-actin, associated with an improved G-actin level in hepatocyte cell line by PTX-1, PTX-2, and PTX-11 (1–1000 nM) treatments, was observed via confocal image analysis. However, no activity was observed by treatment with PTX-2 seco acid (PTX-2 SA), which is an enzymatically digested derivative of PTX-2 [105]. PTXs were initially classified into the DSP-producing group; however, mice toxicity tests confirmed that this toxin does not induce diarrheic symptoms but causes severe hepatotoxicity [106]. Specifically, Miles et al. [107] previously developed an effective method to isolate pectenotoxins from dinoflagellate cells, and they showed isolated PTX-2 caused acute toxicity in mice, whereas its derivative, PTX-2 SA, had no effect at 5000 µg/kg. Additionally, no diarrhea was observed in mice receiving either PTX-2 or PTX-2 SA treatments [107]. The structures and analogues of PTXs were previously described by Allingham et al. [76] and Wilkins et al. [108]. OA can be produced by dinoflagellates *D. acuta*, *D. acuminate*, *D. fortii*, *Prorocentrum concavum*, *P. rhathymum*, *P. belizeanum*, *P. lima*, and *P. arenarium* [109,110,111,112]. DPXs are produced by *D. acuta*, *D. acuminate*, *P. foraminosum*, and *P. lima* [93,113,114,115], whereas PTXs are produced by *D. fortii*, *D. acuta*, *D. acuminate*, and *D. caudata* [109,116,117].

*Gambierdiscus toxicus* produces ciguatera-inducing maitotoxin (MTX) [118]. Holmes and Lewisk purified *G. toxicus*-derived distinct MTXs using high-pressure liquid chromatography and reported that these compounds caused contractile responses of the muscle [108]. MTX is considered one of the largest natural products (3422 Da) that can activate cellular calcium channels [79,119]. Takahashi et al. [79] showed the association of increased calcium influx and calcium-dependent release of [3H] norepinephrine in a pheochromocytoma cell line. Their findings indicated that MTX’s mode of action is the activation of cellular calcium channels. The structures and analogues were previously described by Reyes et al. [120].

*Karenia* spp. dinoflagellates, including *K. brevis* and *K. mikimotoi*, are well-recognized as harmful algae in Japan and the USA [113,114]; these species produce brevetoxin (PbTx) and gymnocin (GC), respectively [85,86,87]. PbTx activates mammalian voltage-gated sodium channels, thereby causing NSP [20,84,121]. Further, aerosolized PbTx in sea spray causes reduced respiratory function and asthma [116]. The structures, analogues, and toxicity of PbTx were previously well-elucidated by European Food Safety Authority (EFSA) panels [122]. GCs are polyether toxins that include GC-A and GC-B [86,87]. Although their modes of action are still poorly understood, GCs are carboxylic acids and show moderate cytotoxicity activity against mouse lymphoid P388 cells [86,87]. The structures of GC-A and gymnocin-B were determined by Satake et al. [86,123]. Additionally, Tanaka et al. [87] determined the structures of GC analogues, including GC-A carboxylic acid and GC-A2.

*Ostreopsis* spp. are well-recognized, harmful algae worldwide due to their spread to many tropical and temperate regions. They produce aerosolized palytoxin (PLTX) along with its analogues, which have caused myalgia, respiratory problems, impairment of the neuromuscular apparatus, and abnormalities in cardiac function [124,125]. PLTX is considered one of the most lethal marine toxins, and its mode of action is unique wherein it causes the Na^+^/K^+^ pump to turn into a shape that allows the passive transport of sodium and potassium ions [35,91]. PLTXs and their analogues can be produced by the dinoflagellates *O. siamensis*, *O. ovata,* and *O. mascarenensis* [126,127,128]. The structure, analogues, and toxicity of PLTXs were previously represented by Ramos and Vasconcelos [129].

*Prorocentrum* spp. produce species-specific diverse phycotoxins, such as OA, DPX, prorocentrolides (PC), and borbotoxin (BTX) (Table 1). PCs are a member of the cyclic imine phycotoxins family produced by *P. lima* and *P. maculosum*, and they act on both muscle and neuronal nicotinic acetylcholine receptors (nAChRs) [94,130,131]. *P. borbonicum* produces BTX-A that was purified by Ten-Hage et al. [95] and has a similar mode of action on nAChRs. The general structure, analogues, and modes of action of PCs and BTX were reported by Amar et al. [94] and Ten-Hage et al. [95].

## 4. Potential Biotechnological Applications of Phycotoxins Based on Their Modes of Action

As shown in Table 2, the aforementioned diverse phycotoxins have extensive biotechnological applications in industry, especially for therapeutics and drug development. As described in the previous section, AZAs block the hERG potassium channel [57]. A variety of potassium channel inhibitors are used for the treatment of cardiac arrhythmias with irregular heartbeat [132]. Therefore, AZAs are potential candidates for the development of antiarrhythmic drugs. BTX and PCs demonstrated nAChRs blocking activity; thus, this mechanism can be a potent target for anticholinergic drugs, including nicotinic antagonists [133]. PbTx is being used for research purposes; this toxin is an activator of the voltage-gated sodium channel and can be further evaluated for the development of post-stroke recovery drugs [134]. Sequeira et al. [134] reported that PbTx treatment in mice decreased the percentage of foot faults with a two-fold increase in dendritic arbor complexity. Additionally, they reported that PbTx-2 enhanced the dendritic arborization and synapse density of the cortical layer V pyramidal neurons in the peri-infarct cortex and improved motor recovery [135]. These results demonstrate that PbTx could be a potential candidate for the development of post-stroke recovery drugs. CTX exhibits inhibition of unmyelinated nerves that are related to pain; hence, it can be potentially used in the development of potent analgesics [65]. Sodium channels are highly associated with pain and depressive disorder symptoms [136,137]. For instance, Manríquez et al. [138] previously performed clinical treatment on five bladder pain syndrome (BPS) patients using NSTX, and all five patients successfully responded to the treatment. Furthermore, the analgesic effect of NSTX lasted for 90 days without adverse effects. Therefore, the sodium channel blockers, namely STX, GTX, and NSTX, can be potential candidates for the development of analgesic and antidepressant drugs. 

DPX, OA, and PLTXs are considered tumor promoters. Fujiki et al. [102] previously reported that DPX-1 and OA showed tumor-promoting activities through different pathways in a two-stage carcinogenesis experiment in mouse skin. PLTX is considered a novel tumor promoter in mouse skin, which can be used for studying signaling mechanisms involved in carcinogenesis [149]. Although dinoflagellate-derived HL is not a well-defined phycotoxin, the potential use of HLs as anticancer agents has been previously reported in the case of α-HL produced by the bacterium *Staphylococcus aureus* [143]. Furthermore, Cho et al. [142] reported that the co-culture of dinoflagellate *H. circularisquama* with green microalga *Dunaliella salina* showed effective flocculation activity, which is applicable for harvesting industrially available microalgae in biodiesel production. These results indicated that HLs are potential bioflocculation agents. KmTx is regarded as an allopathic compound; it is a type of HL generated by *Karlodinium* spp., and it exhibited copepod-killing activity [144]. Since KmTx causes cell membrane disruption by binding specifically to cholesterol, this toxin can be a useful component for anticancer or microalgae-harvesting agents, similar to the dinoflagellate HLs, as described above [142,143]. In addition, KTX affects the copepod *Acartia tonsa*, a natural grazer of *Karlodinium* spp. Although the mode of action of KTX has not been studied specifically, this toxin showed massive potential as a piscicide and copepod-killing agent owing to its ichthyotoxicity and toxicity in copepods, respectively. MTX has attracted much attention due to its bioactivity, which involves disruption of cellular calcium homeostasis [120]. Reyes et al. [120] previously described in detail the biomedical applications of MTXs, wherein they proposed MTXs as a tool for analyzing ion channels and insulin secretion, interleukin-1β secretagogue and oncotic death induction, and sperm acrosome reaction induction [120]. OA is a selective inhibitor of the protein phosphatases PP1 and PP2A; thus, it can be used as a powerful probe for the study of regulatory mechanisms and neurotoxicity. Therefore, OA cannot only be used as a tumor promoter, but it can also be instrumental in neurodegeneration studies, including Alzheimer’s disease [72,146,147]. PLTX can be used in research for anticancer drugs. Görögh et al. [148] previously found that PLTX possesses preferential toxicity for head and neck carcinoma cells, with extensive destruction of xenografted tumors within 24 days. Thus, it seems that PLTX plays contrary dual roles in tumor promotion and suppression. In addition, other phycotoxins including PTX, PCs, and YTXs demonstrated potential anticancer activity. According to a previous review conducted by Kim et al. [150], PTX-2 showed significant cytotoxicity in human cancer cells via downregulation of antiapoptotic Bcl-2, IAP-related proteins, upregulation of Bax protein and ligand (TRAIL)-receptor 1/receptor 2 (DR4/DR5), mitochondrial dysfunction, and apoptosis through suppression of the nuclear factor κB pathway. Additionally, in their recent study, Lee et al. [152] reported that prorocentrolide C from cultured dinoflagellate *P. lima* could induce human cancer cell death via apoptosis and cell cycle arrest. Furthermore, YTXs represented anticancer activity in a B16F10 melanoma preclinical mouse model, along with antiallergic activity [155].

As described above, many studies have revealed the potential application of the phycotoxins as therapeutic agents. However, more mechanistic and clinical studies are required for their safe use in humans. Although the review does not encompass all the phycotoxins, we expect that this comprehensive summary will provide useful information and garner much attention for the industrial applications of phycotoxins.

## 5. Conclusions

Over the past few decades, HAB-forming dinoflagellates have been recognized as an environmental, economic, and health problem owing to their adverse effects on the aquatic ecosystem, fishing industry, and human health. In this review, we described the biotechnological potential of diverse dinoflagellate phycotoxins based on the toxins’ intrinsic modes of action. Many studies have reported the potential application of these toxins, especially in medicine. Since algal cells can be easily cultivated in large photobioreactors or open pond systems that sequestrate atmospheric carbon dioxide, the bioproduction of value-added phycotoxins is economically feasible and beneficial to humans and the environment. However, further mechanistic and clinical studies are required for determining the application of dinoflagellate phycotoxins in therapeutics and other fields.

## Figures and Tables

**Table 1:** List of phycotoxins derived from diverse dinoflagellates and their modes of action.

Dinoflagellate	Toxins	Mode of Action	References
*Alexandrium* spp.	Saxitoxin (STX)	Inhibits sodium channel	[34,35]
	Gonyautoxin (GTX)	Inhibits sodium channel	[33,35,36]
	Neosaxitoxin (NSTX)	Inhibits sodium channel	[35,36]
	Haemolysin (HL)	Hydrolyses phospholipids in the bilayer	[36,42]
		Form pores in phospholipid bilayers	
*Amphidinium carterae*	Haemolysin (HL)	Hydrolyses phospholipids in the bilayer	[33,36,45]
		Forms pores in phospholipid bilayers	
*Azadinium spinosum*	Azaspiracids (AZA)	Blocks hERG (human ether-a-go-go related gene) potassium channel by binding to it	[49]
*Cochlodinium polykrikoides*	Haemolysin (HL)	Hydrolyses phospholipids in the bilayer	[36,47,64]
		Forms pores in phospholipid bilayers	
*Coolia* spp.	Cooliatoxin (CTX)	Blocks unmyelinated nerves	[36,65]
	Yessotoxin (YTX)	Activates calcium channel	[66,67,68,69]
		Decreases cytosolic 3′,5′-cyclic adenosine monophosphate (cAMP) levels	
*Dinophysis* spp.	Okadaic acid (OA)	Inhibits protein phosphatases (serine/threonine phosphatases)	[70,71,72,73,74]
	Dinophysistoxin (DPX)	Inhibits protein phosphatases (serine/threonine phosphatases)	[75]
	Pectenotoxin (PTX)	Inhibits protein phosphatases (serine/threonine phosphatases)	[35,76,77,78]
		Depolymerizes actin filaments	
		Disrupts actin cytoskeleton	
*Gambierdiscus toxicus*	Maitotoxin (MTX)	Activates calcium channel	[79]
*Gonyaulax* spp.	Haemolysin (HL)	Hydrolyses phospholipids in the bilayer	[46]
		Forms pores in phospholipid bilayers	
	Yessotoxin (YTX)	Activates calcium channel	[68,69,80]
		Decreases cytosolic 3′,5′-cyclic adenosine monophosphate (cAMP) levels	
*Gymnodinium catenatum*	Saxitoxin (STX)	Inhibits sodium channel	[39]
*Heterocapsa circularisquama*	Haemolysin (HL)	Hydrolyses phospholipids in the bilayer	[48]
		Forms pores in phospholipid bilayers	
*Karlodinium* spp.	Karmitoxin (KTX)	Unknown (Ichthyotoxic)	[81,82]
	Karlotoxin (KmTx)	Disrupts cell membrane by specific binding to cholesterol	[82,83]
*Karenia mikimotoi*	Brevetoxin (PbTx)	Activates voltage-gated sodium channels	[20,84,85]
	Gymnocin (GC)	Unknown	[86,87]
	Haemolysin (HL)	Hydrolyses phospholipids in the bilayer	[88]
		Forms pores in phospholipid bilayers	
*Lingulodinium polyedrum*	Yessotoxin (YTX)	Activates calcium channel	[68,69,89]
		Decreases cytosolic 3′,5′-cyclic adenosine monophosphate (cAMP) levels	
*Ostreopsis* spp.	Palytoxin (PLTX)	Turns Na+/K+ pump into a shape that allows the passive transport of sodium and potassium ions	[36,90,91]
*Prorocentrum* spp.	Okadaic acid (OA)	Inhibits protein phosphatases (serine/threonine phosphatases)	[35,36,92]
		Depolymerizes actin filaments	
		Induces apoptosis through suppression of the nuclear factor ƙB signaling pathway	
	Dinophysistoxin (DPX)	Inhibits protein phosphatase	[75,93]
	Prorocentrolides (PC)	Acts on nicotinic acetylcholine receptors (nAChRs)	[94]
	Borbotoxin (BTX)	Blocks postsynaptic nAChRs	[95]
*Protoceratium reticulatum*	Yessotoxin (YTX)	Activates calcium channel	[68,89]
		Decreases cytosolic adenosine 3′,5′-cyclic monophosphate (cAMP) levels	
*Pyrodinium bahamense*	Saxitoxin (STX)	Blocks sodium channel	[40]

**Table 2:** Industrial use and biotechnological potential of diverse dinoflagellate phycotoxins.

Toxins	Industrial Use	Potential Applications	References
azaspiracids	Unknown	Antiarrhythmic drugs	[57]
borbotoxin	Unknown	Anticholinergic drugs	[95]
brevetoxin	Research	Post-stroke recovery drugs	[134]
cooliatoxin	Unknown	Unmyelinated nerve fiber-related research	[65]
		Analgesics	
dinophysistoxin	Research	Tumor promoter	[102]
gonyautoxin	Treatment against acute or chronic anal fissures, research	Analgesics, anesthetics, and	[139,140]
		antidepressant drugs	
hemolysins	Unknown	Anticancer drugs	[141,142,143]
		Microalgae-harvesting agents	
karlotoxin	Unknown	Copepod-killing agent	[141,142,144]
		Anticancer drugs	
		Microalgae-harvesting agents	
karmitoxin	Unknown	Piscicide	[82]
		Copepod-killing agent	
maitotoxin	Research	As a tool for the analyses of ion channels and insulin secretion	[120]
		Interleukin-1β inducer	
		Sperm acrosome reaction inducer	
neosaxitoxin	Research	Analgesics, anesthetics, and antidepressant drugs	[138,139,145]
		Long-acting pain blocker in bladder pain syndrome	
okadaic acid	Research	Probe for discovery of neurodegeneration drugs	[72,146,147]
		Tumor promoter	
palytoxin	Research	Tumor promoter	[148,149]
		Anticancer drugs	
pectenotoxin	Research	Anticancer drugs	[150,151]
		Anti-actin drugs	
prorocentrolides	Unknown	Anticancer drugs	[152]
		Anticholinergic drugs	
saxitoxin	Chemical weapon, research	Analgesics, anesthetics, and antidepressant drugs	[139,153,154]
yessotoxin	Research	Anticancer drugs	[155]
		Antiallergic drugs	

## References

[1] Heimann K., Huerlimann R. (2015). Microalgal classification: Major classes and genera of commercial microalgal species. Handbook of Marine Microalgae.

[2] Gupta S.K., Ansari F.A., Shriwastav A., Sahoo N.K., Rawat I., Bux F. (2016). Dual role of *Chlorella sorokiniana* and *Scenedesmus obliquus* for comprehensive wastewater treatment and biomass production for bio-fuels. J. Clean. Prod..

[3] Moulton T.P., Borowitzka L.J., Vincent D.J. (1987). The mass culture of *Dunaliella salina* for *β*-carotene: From pilot plant to production plant. Twelfth International Seaweed Symposium.

[4] Priyadarshani I., Rath B. (2012). Commercial and industrial applications of micro algae—A review. J. Algal Biomass Utln..

[5] Pulz O., Gross W. (2004). Valuable products from biotechnology of microalgae. Appl. Microbiol. Biotechnol..

[6] Safi C., Zebib B., Merah O., Pontalier P.Y., Vaca-Garcia C. (2014). Morphology, composition, production, processing and applications of *Chlorella vulgaris*: A review. Renew. Sustain. Energy Rev..

[7] Spolaore P., Joannis-Cassan C., Duran E., Isambert A. (2006). Commercial applications of microalgae. J. Biosci. Bioeng..

[8] Handy S.M., Coyne K.J., Portune K.J., Demir E., Doblin M.A., Hare C.E., Cary S.C., Hutchins D.A. (2005). Evaluating vertical migration behavior of harmful raphidophytes in the Delaware Inland Bays utilizing quantitative real-time PCR. Aquat. Microb. Ecol..

[9] Kim S.H., Kim K.Y., Kim C.H., Lee W.S., Chang M., Lee J.H. (2004). Phylogenetic analysis of harmful algal bloom (HAB)-causing dinoflagellates along the Korean coasts, based on SSU rRNA gene. J. Microbiol. Biotechnol..

[10] Landsberg J.H. (2002). The effects of harmful algal blooms on aquatic organisms. Rev. Fish. Sci..

[11] Sakamoto S., Lim W.A., Lu D., Dai X., Orlova T., Iwataki M. (2020). Harmful algal blooms and associated fisheries damage in East Asia: Current status and trends in China, Japan, Korea and Russia. Harmful Algae.

[12] Wolf D., Georgic W., Klaiber H.A. (2017). Reeling in the damages: Harmful algal blooms’ impact on Lake Erie’s recreational fishing industry. J. Environ. Manag..

[13] Dorantes-Aranda J.J., Seger A., Mardones J.I., Nichols P.D., Hallegraeff G.M. (2015). Progress in understanding algal bloom-mediated fish kills: The role of superoxide radicals, phycotoxins and fatty acids. PLoS ONE.

[14] Kim D., Nakamura A., Okamoto T., Komatsu N., Oda T., Iida T., Ishimatsu A., Muramatsu T. (2000). Mechanism of superoxide anion generation in the toxic red tide phytoplankton *Chattonella marina*: Possible involvement of NAD (P) H oxidase. Biochim. Biophys. Acta Gen. Subj..

[15] Martins J.C., Vasconcelos V.M. (2009). Microcystin dynamics in aquatic organisms. J. Toxicol. Environ. Health B Crit. Rev..

[16] Stoecker D.K. (1999). Mixotrophy among Dinoflagellates 1. J. Eukaryot. Microbiol..

[17] Fraga S., Rodríguez F., Bravo I., Zapata M., Marañón E. (2012). Review of the main ecological features affecting benthic dinoflagellate blooms. Cryptogam. Algol..

[18] Hallegraeff G.M. (1993). A review of harmful algal blooms and their apparent global increase. Phycologia.

[19] Hallegraeff G.M. (2003). Manual on harmful marine microalgae. International Oceanographic Commission Manuals and Guides Number 33.

[20] Wang D.Z. (2008). Neurotoxins from marine dinoflagellates: A brief review. Mar. Drugs.

[21] Gómez F. (2012). A checklist and classification of living dinoflagellates (*Dinoflagellata, Alveolata*). CICIMAR Ocean..

[22] Camacho F.G., Rodríguez J.G., Mirón A.S., García M.C., Belarbi E.H., Chisti Y., Grima E.M. (2007). Biotechnological significance of toxic marine dinoflagellates. Biotechnol. Adv..

[23] Kim H.G. (1997). Recent harmful algal blooms and mitigation strategies in Korea. Ocean. Polar Res..

[24] Kudela R.M., Gobler C.J. (2012). Harmful dinoflagellate blooms caused by *Cochlodinium* sp.: Global expansion and ecological strategies facilitating bloom formation. Harmful Algae.

[25] Chen I.C., Hill J.K., Ohlemüller R., Roy D.B., Thomas C.D. (2011). Rapid range shifts of species associated with high levels of climate warming. Science.

[26] Chang F.H., Gall M. (2013). Pigment compositions and toxic effects of three harmful Karenia species, *Karenia concordia*, *Karenia brevisulcata* and *Karenia mikimotoi* (Gymnodiniales, Dinophyceae), on rotifers and brine shrimps. Harmful Algae.

[27] Samson J.C., Shumway S.E., Weis J.S. (2008). Effects of the toxic dinoflagellate, *Alexandrium fundyense* on three species of larval fish: A food-chain approach. J. Fish. Biol..

[28] Scott A.B. (1980). Botulinum toxin injection into extraocular muscles as an alternative to strabismus surgery. J. Pediatr. Ophthalmol. Strabismus.

[29] Barrientos N., Chana P. (2003). Botulinum toxin type a in prophylactic treatment of migraine headaches: A preliminary study. J. Headache Pain.

[30] Hambleton P. (1992). *Clostridium botulinum* toxins: A general review of involvement in disease, structure, mode of action and preparation for clinical use. J. Neurol..

[31] Song H., Li J., Lu C.L., Kang L., Xie L., Zhang Y.Y., Zhou X.B., Zhong S. (2011). Tetrodotoxin alleviates acute heroin withdrawal syndrome: A multicentre, randomized, double-blind, placebo-controlled study. Clin. Exp. Pharmacol. Physiol..

[32] Hagen N.A., Lapointe B., Ong–Lam M., Dubuc B., Walde D., Gagnon B., Love R., Goel R., Hawley P., Ngoc A.H. (2011). multicentre open-label safety and efficacy study of tetrodotoxin for cancer pain. Curr. Oncol..

[33] Baig H.S., Saifullah S.M., Dar A. (2006). Occurrence and toxicity of *Amphidinium carterae* Hulburt in the North Arabian Sea. Harmful Algae.

[34] Lefebvre K.A., Bill B.D., Erickson A., Baugh K.A., O’Rourke L., Costa P.R., Nance S., Trainer V.L. (2008). Characterization of intracellular and extracellular saxitoxin levels in both field and cultured *Alexandrium* spp. samples from Sequim Bay, Washington. Mar. Drugs.

[35] Stonik V.A., Stonik I.V., Gopalakrishnakone P., Haddad V., Kem W., Tubaro A., Kim E. (2014). Toxins Produced by Marine Microorganisms: A Mini Review. Marine and Freshwater Toxins.

[36] Zingone A., Siano R., D’Alelio D., Sarno D. (2006). Potentially toxic and harmful microalgae from coastal waters of the Campania region (Tyrrhenian Sea, Mediterranean Sea). Harmful Algae.

[37] Durán-Riveroll L.M., Cembella A.D. (2017). Guanidinium toxins and their interactions with voltage-gated sodium ion channels. Mar. Drugs.

[38] Wiese M., D’agostino P.M., Mihali T.K., Moffitt M.C., Neilan B.A. (2010). Neurotoxic alkaloids: Saxitoxin and its analogs. Mar. Drugs.

[39] Sako Y., Yoshida T., Uchida A., Arakawa O., Noguchi T., Ishida Y. (2001). Purification and characterization of a sulfotransferase specific to N-21 of saxitoxin and gonyautoxin 2+ 3 from the toxic dinoflagellate *Gymnodinium catenatum* (Dinophyceae). J. Phycol..

[40] Landsberg J.H., Hall S., Johannessen J.N., White K.D., Conrad S.M., Abbott J.P., Flewelling L.J., Richardson R.W., Dickey R.W., Jester E.L.E. (2006). Saxitoxin puffer fish poisoning in the United States, with the first report of *Pyrodinium bahamense* as the putative toxin source. Environ. Health Perspect..

[41] Andrinolo D., Michea L.F., Lagos N. (1999). Toxic effects, pharmacokinetics and clearance of saxitoxin, a component of paralytic shellfish poison (PSP), in cats. Toxicon.

[42] Tatters A.O., Van Wagoner R.M., Wright J.L., Tomas C.R. (2012). Regulation of spiroimine neurotoxins and hemolytic activity in laboratory cultures of the dinoflagellate *Alexandrium peruvianum* (Balech & Mendiola) Balech & Tangen. Harmful Algae.

[43] Chalmeau J., Monina N., Shin J., Vieu C., Noireaux V. (2011). α-Hemolysin pore formation into a supported phospholipid bilayer using cell-free expression. Biochim. Biophys. Acta Biomembr..

[44] Bhakdi S., Mackman N., Menestrina G., Gray L., Hugo F., Seeger W., Holland I.B. (1988). The hemolysin of *Escherichia coli*. Eur. J. Epidemiol..

[45] Pagliara P., Caroppo C. (2012). Toxicity assessment of *Amphidinium carterae*, *Coolia* cfr. *monotis* and *Ostreopsis* cfr. *ovata* (Dinophyta) isolated from the northern Ionian Sea (Mediterranean Sea). Toxicon.

[46] Bass E.L., Pinion J.P., Sharif M.E. (1983). Characteristics of a hemolysin from *Gonyaulax monilata* Howell. Aquat. Toxicol..

[47] Dorantes-Aranda J.J., García-de la Parra L.M., Alonso-Rodríguez R., Morquecho L. (2009). Hemolytic activity and fatty acids composition in the ichthyotoxic dinoflagellate *Cochlodinium polykrikoides* isolated from Bahía de La Paz, Gulf of California. Mar. Pollut. Bull..

[48] Oda T., Sato Y., Kim D., Muramatsu T., Matsuyama Y., Honjo T. (2001). Hemolytic activity of *Heterocapsa circularisquama* (Dinophyceae) and its possible involvement in shellfish toxicity. J. Phycol..

[49] Salas R., Tillmann U., John U., Kilcoyne J., Burson A., Cantwell C., Hess P., Jauffrais T., Silke J. (2011). The role of *Azadinium spinosum* (Dinophyceae) in the production of azaspiracid shellfish poisoning in mussels. Harmful Algae.

[50] Satake M., Ofuji K., Naoki H., James K.J., Furey A., McMahon T., Silke J., Yasumoto T. (1998). Azaspiracid, a new marine toxin having unique spiro ring assemblies, isolated from Irish mussels, Mytilus edulis. J. Am. Chem. Soc..

[51] James K.J., Moroney C., Roden C., Satake M., Yasumoto T., Lehane M., Furey A. (2003). Ubiquitous ‘benign’alga emerges as the cause of shellfish contamination responsible for the human toxic syndrome, azaspiracid poisoning. Toxicon.

[52] Botana L.M., Alfonso A., Vale C., Vilariño N., Rubiolo J., Alonso E., Cagide E. (2014). The mechanistic complexities of phycotoxins: Toxicology of azaspiracids and yessotoxins. Advances in Molecular Toxicology.

[53] Klontz K.C., Abraham A., Plakas S.M., Dickey R.W. (2009). Mussel-associated azaspiracid intoxication in the United States. Ann. Intern. Med..

[54] Ito E., Satake M., Ofuji K., Kurita N., McMahon T., James K., Yasumoto T. (2000). Multiple organ damage caused by a new toxin azaspiracid, isolated from mussels produced in Ireland. Toxicon.

[55] Pelin M., Sosa S., Brovedani V., Kilcoyne J., Nulty C., Hess P., Tubaro A. (2016). In vitro effects of three azaspiracid analogues on hepatocytes. Toxicon.

[56] Pelin M., Kilcoyne J., Nulty C., Crain S., Hess P., Tubaro A., Sosa S. (2018). Toxic equivalency factors (TEFs) after acute oral exposure of azaspiracid 1,− 2 and− 3 in mice. Toxicol. Lett..

[57] Twiner M.J., Doucette G.J., Rasky A., Huang X.P., Roth B.L., Sanguinetti M.C. (2012). Marine algal toxin azaspiracid is an open-state blocker of hERG potassium channels. Chem. Res. Toxicol..

[58] Pelin M., Kilcoyne J., Florio C., Hess P., Tubaro A., Sosa S. (2019). Azaspiracids increase mitochondrial dehydrogenases activity in hepatocytes: Involvement of potassium and chloride ions. Mar. Drugs.

[59] Hess P., Twiner J.M., Kilcoyne J., Sosa S. (2015). Azaspiracid toxins: Toxicological profile. Marine and Freshwater Toxins.

[60] Kilcoyne J., Twiner M.J., McCarron P., Crain S., Giddings S.D., Foley B., Rise F., Hess P., Wilkins A.L., Miles C.O. (2015). Structure elucidation, relative LC–MS response and in vitro toxicity of azaspiracids 7–10 isolated from mussels (*Mytilus edulis*). J. Agric. Food Chem..

[61] Kilcoyne J., McCarron P., Twiner M.J., Rise F., Hess P., Wilkins A.L., Miles C.O. (2018). Identification of 21, 22-dehydroazaspiracids in mussels (*Mytilus edulis*) and in vitro toxicity of azaspiracid-26. J. Nat. Prod..

[62] Ofuji K., Satake M., McMahon T., JAMES K.J., Naoki H., Oshima Y., Yasumoto T. (2001). Structures of azaspiracid analogs, azaspiracid-4 and azaspiracid-5, causative toxins of azaspiracid poisoning in Europe. Biosci. Biotechnol. Biochem..

[63] Twiner M.J., Hess P., Doucette G.J. (2014). Azaspiracids: Toxicology, Pharmacologiy, and Risk Assessment. Seafood and Freshwater Toxins.

[64] Kim C.S., Lee S.G., Kim H.G., Lee J.S. (2001). Screening for toxic compounds in the red tide dinoflagellate *Cochlodinium polykrikoides*: Is it toxic plankton?. ALGAE.

[65] Holmes M.J., Lewis R.J., Jones A., Hoy A.W.W. (1995). Cooliatoxin, the first toxin from *Coolia monotis* (Dinophyceae). Nat. Toxins.

[66] Wakeman K.C., Yamaguchi A., Roy M.C., Jenke-Kodama H. (2015). Morphology, phylogeny and novel chemical compounds from *Coolia malayensis* (Dinophyceae) from Okinawa, Japan. Harmful Algae.

[67] de Azevedo Tibiriga C.E.J., Sibat M., Fernandes L.F., Bilien G., Chomerat N., Hess P., Mafra L.L. (2020). Diversity and Toxicity of the Genus *Coolia* Meunier in Brazil, and Detection of 44-methyl Gambierone in *Coolia tropicalis*. Toxins.

[68] de la Rosa L.A., Alfonso A., Vilariño N., Vieytes M.R., Botana L.M. (2001). Modulation of cytosolic calcium levels of human lymphocytes by yessotoxin, a novel marine phycotoxin. Biochem. Pharmacol..

[69] Alfonso A., Vieytes M.R., Botana L.M. (2016). Yessotoxin, a promising therapeutic tool. Mar. Drugs.

[70] Bodero M., Hoogenboom R.L., Bovee T.F., Portier L., de Haan L., Peijnenburg A., Hendriksen P.J. (2018). Whole genome mRNA transcriptomics analysis reveals different modes of action of the diarrheic shellfish poisons okadaic acid and dinophysis toxin-1 versus azaspiracid-1 in Caco-2 cells. Toxicol. In Vitro.

[71] Cohen P., Holmes C.F., Tsukitani Y. (1990). Okadaic acid: A new probe for the study of cellular regulation. Trends Biochem. Sci..

[72] Kamat P.K., Rai S., Swarnkar S., Shukla R., Nath C. (2014). Molecular and cellular mechanism of okadaic acid (OKA)-induced neurotoxicity: A novel tool for Alzheimer’s disease therapeutic application. Mol. Neurobiol..

[73] Reguera B., Riobó P., Rodríguez F., Díaz P.A., Pizarro G., Paz B., José M.F., Blanco J. (2014). Dinophysis toxins: Causative organisms, distribution and fate in shellfish. Mar. Drugs.

[74] Sathasivam R., Radhakrishnan R., Hashem A., Abd_Allah E.F. (2019). Microalgae metabolites: A rich source for food and medicine. Saudi J. Biol. Sci..

[75] Vale C., Botana L.M. (2008). Marine toxins and the cytoskeleton: Okadaic acid and dinophysistoxins. FEBS J..

[76] Allingham J.S., Miles C.O., Rayment I. (2007). A structural basis for regulation of actin polymerization by pectenotoxins. J. Mol. Biol..

[77] Basti L., Nagai S., Go J., Okano S., Nagai K., Watanabe R., Suzuki T., Tanaka Y. (2015). Differential inimical effects of *Alexandrium* spp. and *Karenia* spp. on cleavage, hatching, and two larval stages of Japanese pearl oyster *Pinctada fucata martensii*. Harmful Algae.

[78] Rossini G.P., Hess P., Luch A. (2010). Phycotoxins: Chemistry, mechanisms of action and shellfish poisoning. Molecular, Clinical and Environmental Toxicology. Volume2: Clinical Toxicology.

[79] Takahashi M., Ohizumi Y., Yasumoto T. (1982). Maitotoxin, a Ca^2+^ channel activator candidate. J. Biol. Chem..

[80] Rhodes L., McNabb P., De Salas M., Briggs L., Beuzenberg V., Gladstone M. (2006). Yessotoxin production by *Gonyaulax spinifera*. Harmful Algae.

[81] Andersen A.J.C., De Medeiros L.S., Binzer S.B., Rasmussen S.A., Hansen P.J., Nielsen K.F., Jørgensen K., Larsen T.O. (2017). HPLC-HRMS Quantification of the Ichthyotoxin Karmitoxin from *Karlodinium armiger*. Mar. Drugs.

[82] Rasmussen S.A., Binzer S.B., Hoeck C., Meier S., De Medeiros L.S., Andersen N.G., Place A., Nielsen K.F., Hansen P.J., Larsen T.O. (2017). Karmitoxin: An amine-containing polyhydroxy-polyene toxin from the marine dinoflagellate *Karlodinium armiger*. J. Nat. Prod..

[83] Van Wagoner R.M., Deeds J.R., Satake M., Ribeiro A.A., Place A.R., Wright J.L. (2008). Isolation and characterization of karlotoxin 1, a new amphipathic toxin from *Karlodinium veneficum*. Tetrahedron Lett..

[84] Bourdelais A.J., Campbell S., Jacocks H., Naar J., Wright J.L., Carsi J., Baden D.G. (2004). Brevenal is a natural inhibitor of brevetoxin action in sodium channel receptor binding assays. Cell. Mol. Neurobiol..

[85] Novoveská L., Robertson A. (2019). Brevetoxin-Producing Spherical Cells Present in Karenia brevis Bloom: Evidence of Morphological Plasticity?. J. Mar. Sci. Eng..

[86] Satake M., Tanaka Y., Ishikura Y., Oshima Y., Naoki H., Yasumoto T. (2005). Gymnocin-B with the largest contiguous polyether rings from the red tide dinoflagellate, *Karenia* (formerly *Gymnodinium*) *mikimotoi*. Tetrahedron Lett..

[87] Tanaka Y., Satake M., Yotsu-Yamashita M., Oshima Y. (2013). Gymnocin-A carboxylic acid and gymnocin-A2, cytotoxic polyethers from the red tide dinoflagellate *Karenia mikimotoi*. Heterocycles.

[88] Kim D., Li W., Matsuyama Y., Matsuo A., Yagi M., Cho K., Yamasaki Y., Takeshita S., Yamaguchi K., Oda T. (2020). Strain-dependent lethal effects on abalone and haemolytic activities of the dinoflagellate *Karenia mikimotoi*. Aquaculture.

[89] Paz B., Riobó P., Fernández M.L., Fraga S., Franco J.M. (2004). Production and release of yessotoxins by the dinoflagellates *Protoceratium reticulatum* and *Lingulodinium polyedrum* in culture. Toxicon.

[90] Ciminiello P., Dell’Aversano C., Iacovo E.D., Fattorusso E., Forino M., Tartaglione L., Benedettini G., Onorari M., Serena F., Battocchi C. (2014). First finding of *Ostreopsis* cf. *ovata* toxins in marine aerosols. Environ. Sci. Technol..

[91] Wu C.H. (2009). Palytoxin: Membrane mechanisms of action. Toxicon.

[92] Campos A., Freitas M., de Almeida A.M., Martins J.C., Domínguez-Pérez D., Osório H., Vasconcelos V., Costa P.R. (2020). OMICs Approaches in Diarrhetic Shellfish Toxins Research. Toxins.

[93] Kameneva P.A., Efimova K.V., Rybin V.G., Orlova T.Y. (2015). Detection of dinophysistoxin-1 in clonal culture of marine dinoflagellate *Prorocentrum foraminosum* (Faust MA, 1993) from the Sea of Japan. Toxins.

[94] Amar M., Aráoz R., Iorga B.I., Yasumoto T., Servent D., Molgó J. (2018). Prorocentrolide-A from cultured *Prorocentrum lima* dinoflagellates collected in Japan blocks sub-types of nicotinic acetylcholine receptors. Toxins.

[95] Ten-Hage L., Robillot C., Turquet J., Le Gall F., Le Caer J.P., Bultel V., Guyot M., Molgó J. (2002). Effects of toxic extracts and purified borbotoxins from *Prorocentrum borbonicum* (Dinophyceae) on vertebrate neuromuscular junctions. Toxicon.

[96] Paz B., Daranas A.H., Norte M., Riobó P., Franco J.M., Fernández J.J. (2008). Yessotoxins, a group of marine polyether toxins: An overview. Mar. Drugs.

[97] Binzer S.B., Varga E., Andersen A.J.C., Svenssen D.K., De Medeiros L.S., Rasmussen S.A., Larsen T.O., Hansen P.J. (2020). Karmitoxin production by *Karlodinium armiger* and the effects of *K. armiger* and karmitoxin towards fish. Harmful Algae.

[98] Van Wagoner R.M., Deeds J.R., Tatters A.O., Place A.R., Tomas C.R., Wright J.L. (2010). Structure and relative potency of several karlotoxins from *Karlodinium veneficum*. J. Nat. Prod..

[99] Murata M., Sano M., Iwashita T., Naoki H., YasuMoto T. (1986). The structure of pectenotoxin-3, a new constituent of diarrhetic shellfish toxins. Agric. Biol. Chem..

[100] Twiner M.J., Doucette G.J., Pang Y., Fang C., Forsyth C.J., Miles C.O. (2016). Structure–activity relationship studies using natural and synthetic okadaic acid/dinophysistoxin toxins. Mar. Drugs.

[101] Arias C., Becerra-García F., Arrieta I., Tapia R. (1998). The Protein Phosphatase Inhibitor Okadaic Acid Induces Heat Shock Protein Expression and Neurodegeneration in Rat Hippocampusin Vivo. Exp. Neurol..

[102] Fujiki H., Suganuma M., Suguri H., Yoshizawa S., Takagi K., Uda N., Wakamatsu K., Yamada K., Murata M., Yasumoto T. (1988). Diarrhetic shellfish toxin, dinophysistoxin-1, is a potent tumor promoter on mouse skin. Jpn. J. Cancer Res..

[103] Uchida H., Watanabe R., Matsushima R., Oikawa H., Nagai S., Kamiyama T., Matsuyama Y. (2018). Toxin profiles of okadaic acid analogues and other lipophilic toxins in Dinophysis from Japanese coastal waters. Toxins.

[104] Fernandez J.J., Candenas M.L., Souto M.L., Trujillo M.M., Norte M. (2002). Okadaic acid, useful tool for studying cellular processes. Curr. Med. Chem..

[105] Espina B., Louzao M.C., Ares I.R., Cagide E., Vieytes M.R., Vega F.V., Rubiolo J.A., Miles C.O., Suzuki T., Yasumoto T. (2008). Cytoskeletal toxicity of pectenotoxins in hepatic cells. Br. J. Pharmacol..

[106] Espiña B., Rubiolo J.A. (2008). Marine toxins and the cytoskeleton: Pectenotoxins, unusual macrolides that disrupt actin. FEBS J..

[107] Miles C.O., Wilkins A.L., Munday R., Dines M.H., Hawkes A.D., Briggs L.R., Sandvik M., Jensen D.J., Cooney J.M., Holland P.T. (2004). Isolation of pectenotoxin-2 from *Dinophysis* acuta and its conversion to pectenotoxin-2 seco acid, and preliminary assessment of their acute toxicities. Toxicon.

[108] Wilkins A.L., Rehmann N., Torgersen T., Rundberget T., Keogh M., Petersen D., Hess P., Rise F., Miles C.O. (2006). Identification of fatty acid esters of pectenotoxin-2 seco acid in blue mussels (*Mytilus edulis*) from Ireland. J. Agric. Food Chem..

[109] MacKenzie L., Beuzenberg V., Holland P., McNabb P., Suzuki T., Selwood A. (2005). Pectenotoxin and okadaic acid-based toxin profiles in *Dinophysis acuta* and *Dinophysis acuminata* from New Zealand. Harmful Algae.

[110] Dickey R.W., Bobzin S.C., Faulkner D.J., Bencsath F.A., Andrzejewski D. (1990). Identification of okadaic acid from a Caribbean dinoflagellate, *Prorocentrum concavum*. Toxicon.

[111] An T., Winshell J., Scorzetti G., Fell J.W., Rein K.S. (2010). Identification of okadaic acid production in the marine dinoflagellate *Prorocentrum rhathymum* from Florida Bay. Toxicon.

[112] Ten-Hage L., Delaunay N., Pichon V., Couté A., Puiseux-Dao S., Turquet J. (2000). Okadaic acid production from the marine benthic dinoflagellate *Prorocentrum arenarium* Faust (Dinophyceae) isolated from Europa Island coral reef ecosystem (SW Indian Ocean). Toxicon.

[113] Nielsen L.T., Krock B., Hansen P.J. (2013). Production and excretion of okadaic acid, pectenotoxin-2 and a novel dinophysistoxin from the DSP-causing marine dinoflagellate *Dinophysis acuta–*effects of light, food availability and growth phase. Harmful Algae.

[114] Kim T., Suzuki T. (2009). Production of dinophysistoxin-1 and pectenotoxin-2 by a culture of *Dinophysis acuminata* (Dinophyceae). Harmful Algae.

[115] Jeffrey L.C. (1995). Identification of DTX-4, a new water-soluble phosphatase inhibitor from the toxic dinoflagellate *Prorocentrum lima*. J. Chem. Soc. Chem. Commun..

[116] Suzuki T., Mitsuya T., Matsubara H., Yamasaki M. (1998). Determination of pectenotoxin-2 after solid-phase extraction from seawater and from the dinoflagellate *Dinophysis fortii* by liquid chromatography with electrospray mass spectrometry and ultraviolet detection: Evidence of oxidation of pectenotoxin-2 to pectenotoxin-6 in scallops. J. Chromatogr. A.

[117] Suzuki T., Beuzenberg V., Mackenzie L., Quilliam M.A. (2003). Liquid chromatography–mass spectrometry of spiroketal stereoisomers of pectenotoxins and the analysis of novel pectenotoxin isomers in the toxic dinoflagellate Dinophysis acuta from New Zealand. J. Chromatogr..

[118] Holmes M.J., Lewis R.J. (1994). Purification and characterisation of large and small maitotoxins from cultured *Gambierdiscus toxicus*. Nat. Toxins.

[119] Murata M., Naoki H., Iwashita T., Matsunaga S., Sasaki M., Yokoyama A., Yasumoto T. (1993). Structure of maitotoxin. J. Am. Chem. Soc..

[120] Reyes J.G., Sánchez-Cárdenas C., Acevedo-Castillo W., Leyton P., López-González I., Felix R., Gandini M.A., Treviño M.B., Treviño C.L., Botana L.M. (2014). Maitotoxin: An enigmatic toxic molecule with useful applications in the biomedical sciences. Seafood and Freshwater Toxins: Pharmacology, Physiology and Detection.

[121] Morohashi A., Satake M., Naoki H., Kaspar H.F., Oshima Y., Yasumoto T. (1999). Brevetoxin B4 isolated from greenshell mussels *Perna canaliculus*, the major toxin involved in neurotoxic shellfish poisoning in New Zealand. Nat. Toxins.

[122] EFSA Panel on Contaminants in the Food Chain (CONTAM) (2010). Scientific Opinion on marine biotoxins in shellfish–Emerging toxins: Brevetoxin group. EFSA J..

[123] Satake M., Shoji M., Oshima Y., Naoki H., Fujita T., Yasumoto T. (2002). Gymnocin-A, a cytotoxic polyether from the notorious red tide dinoflagellate, *Gymnodinium mikimotoi*. Tetrahedron Lett..

[124] Pistocchi R., Pezzolesi L., Guerrini F., Vanucci S., Dell’Aversano C., Fattorusso E. (2011). A review on the effects of environmental conditions on growth and toxin production of *Ostreopsis ovata*. Toxicon.

[125] Tubaro A., Durando P., Del Favero G., Ansaldi F., Icardi G., Deeds J.R., Sosa S. (2011). Case definitions for human poisonings postulated to palytoxins exposure. Toxicon.

[126] Usami M., Satake M., Ishida S., Inoue A., Kan Y., Yasumoto T. (1995). Palytoxin analogs from the din1flagellate *Ostreopsis siamensis*. J. Am. Chem. Soc..

[127] Lenoir S., Ten-Hage L., Turquet J., Quod J.P., Bernard C., Hennion M.C. (2004). First evidence of palytoxin analogues from an *Ostreopsis mascarenensis* (dinophyceae) benthic bloom in southwestern indian Ocean 1. J. Phycol..

[128] Bravo I., Vila M., Casabianca S., Rodriguez F., Rial P., Riobó P., Penna A. (2012). Life cycle stages of the benthic palytoxin-producing dinoflagellate *Ostreopsis* cf. *ovata* (Dinophyceae). Harmful Algae.

[129] Ramos V., Vasconcelos V. (2010). Palytoxin and analogs: Biological and ecological effects. Mar. Drugs.

[130] Torigoe K., Murata M., Yasumoto T., Iwashita T. (1988). Prorocentrolide, a toxic nitrogenous macrocycle from a marine dinoflagellate, *Prorocentrum lima*. J. Am. Chem. Soc..

[131] Hu T., DeFreitas A.S., Curtis J.M., Oshima Y., Walter J.A., Wright J.L. (1996). Isolation and structure of prorocentrolide B, a fast-acting toxin from *Prorocentrum maculosum*. J. Nat. Prod..

[132] Colatsky T.J., Follmer C.H., Starmer C.F. (1990). Channel specificity in antiarrhythmic drug action. Mechanism of potassium channel block and its role in suppressing and aggravating cardiac arrhythmias. Circulation.

[133] Gao Z.G., Liu B.Y., Cui W.Y., Li L.J., Fan Q.H., Liu C.G. (1998). Pharmacology: Anti-nicotinic Properties of Anticholinergic Antiparkinson Drugs. J. Pharm. Pharmacol..

[134] Sequeira E., Pierce M., Gomez D., Murray T. (2019). Investigation of the Potential Therapeutic Use of Brevetoxin to Promote Ischemic Stroke Recovery. FASEB J..

[135] Sequeira E., Pierce M.L., Akasheh D., Sellers S., Gerwick W.H., Baden D.G., Murray T.F. (2020). Epicortical Brevetoxin Treatment Promotes Neural Repair and Functional Recovery after Ischemic Stroke. Mar. Drugs.

[136] Waxman S.G., Dib-Hajj S., Cummins T.R., Black J.A. (1999). Sodium channels and pain. Proc. Natl. Acad. Sci. USA.

[137] Bourin M., Chenu F., Hascoet M. (2009). The role of sodium channels in the mechanism of action of antidepressants and mood stabilizers. Curr. Drug Targets.

[138] Manríquez V., Caperan D.C., Guzmán R., Naser M., Iglesia V., Lagos N. (2015). First evidence of neosaxitoxin as a long-acting pain blocker in bladder pain syndrome. Int. Urogynecol. J..

[139] Dick I.E., Brochu R.M., Purohit Y., Kaczorowski G.J., Martin W.J., Priest B.T. (2007). Sodium channel blockade may contribute to the analgesic efficacy of antidepressants. J. Pain.

[140] Garrido R., Lagos N., Lattes K., Abedrapo M., Bocic G., Cuneo A., Chiong H., Jensen C., Azolas R., Henriquez A. (2005). Gonyautoxin: New treatment for healing acute and chronic anal fissures. Dis. Colon Rectum.

[141] Alizadeh S., Barzegari A., Esmaeili A., Omidi Y. (2020). Designing a light-activated recombinant alpha hemolysin for colorectal cancer targeting. Bioimpacts.

[142] Cho K., Hur S.P., Lee C.H., Ko K., Lee Y.J., Kim K.N., Kim M.S., Chung Y.H., Kim D., Oda T. (2016). Bioflocculation of the oceanic microalga *Dunaliella salina* by the bloom-forming dinoflagellate *Heterocapsa circularisquama*, and its effect on biodiesel properties of the biomass. Bioresour. Technol..

[143] Swofford C.A., St. Jean A.T., Panteli J.T., Brentzel Z.J., Forbes N.S. (2014). Identification of *Staphylococcus aureus* α-hemolysin as a protein drug that is secreted by anticancer bacteria and rapidly kills cancer cells. Biotechnol. Bioeng..

[144] Waggett R.J., Tester P.A., Place A.R. (2008). Anti-grazing properties of the toxic dinoflagellate *Karlodinium veneficum* during predator–prey interactions with the copepod *Acartia tonsa*. Mar. Ecol. Prog. Ser..

[145] Rodriguez-Navarro A.J., Lagos N., Lagos M., Braghetto I., Csendes A., Hamilton J., Figueroa C., Truan D., Garcia C., Rojas A. (2007). Neosaxitoxin as a local anestheticpreliminary observations from a first human trial. Anesthesiology.

[146] Forsyth C.J., Dounay A.B., Sabes S.F., Urbanek R.A. (2000). Biotherapeutic Potential and Synthesis of Okadaic Acid. Ernst Schering Research Foundation Workshop Seriese, Volume 32, The Role of Natural Products in Drug Discovery.

[147] Medina M., Avila J., Villanueva N. (2013). Use of okadaic acid to identify relevant phosphoepitopes in pathology: A focus on neurodegeneration. Mar. Drugs.

[148] Görögh T., Bèress L., Quabius E.S., Ambrosch P., Hoffmann M. (2013). Head and neck cancer cells and xenografts are very sensitive to palytoxin: Decrease of c-jun n-terminale kinase-3 expression enhances palytoxin toxicity. Mol. Cancer.

[149] Wattenberg E.V. (2007). Palytoxin: Exploiting a novel skin tumor promoter to explore signal transduction and carcinogenesis. Am. J. Physiol. Cell Physiol..

[150] Kim G.Y., Kim W.J., Choi Y.H. (2011). Pectenotoxin-2 from marine sponges: A potential anti-cancer agent—A review. Mar. Drugs.

[151] Spector I., Braet F., Shochet N.R., Bubb M.R. (1999). New anti-actin drugs in the study of the organization and function of the actin cytoskeleton. Microsc. Res. Tech..

[152] Lee S.M., Kim N.H., Jeong E.J., Rho J.R. (2020). Cytotoxic 4-hydroxyprorocentrolide and prorocentrolide C from cultured Dinoflagellate *Prorocentrum lima* induce human cancer cell death through apoptosis and cell cycle arrest. Toxins.

[153] Adams H.J., Blair Jr M.R., Takman B.H. (1976). The local anesthetic activity of saxitoxin alone and with vasoconstrictor and local anesthetic agents. Arch. Int. Pharmacodyn. Ther..

[154] Stewart C.E. (2006). Weapons of Mass Casualties and Terrorism Response Handbook.

[155] Tobío A., Alfonso A., Madera-Salcedo I., Botana L.M., Blank U. (2016). Yessotoxin, a marine toxin, exhibits anti-allergic and anti-tumoural activities inhibiting melanoma tumour growth in a preclinical model. PLoS ONE.

